# Crop Species Diversity Changes in the United States: 1978–2012

**DOI:** 10.1371/journal.pone.0136580

**Published:** 2015-08-26

**Authors:** Jonathan Aguilar, Greta G. Gramig, John R. Hendrickson, David W. Archer, Frank Forcella, Mark A. Liebig

**Affiliations:** 1 Southwest Research and Extension Center, Kansas State University, Garden City, Kansas, United States of America; 2 Department of Plant Sciences, North Dakota State University, Fargo, North Dakota, United States of America; 3 Northern Great Plains Research Laboratory, Agricultural Research Service, United States Department of Agriculture, Mandan, North Dakota, United States of America; 4 North Central Soil Conservation Research Laboratory, Agricultural Research Service, United States Department of Agriculture, Morris, Minnesota, United States of America; New York State Museum, UNITED STATES

## Abstract

Anecdotal accounts regarding reduced US cropping system diversity have raised concerns about negative impacts of increasingly homogeneous cropping systems. However, formal analyses to document such changes are lacking. Using US Agriculture Census data, which are collected every five years, we quantified crop species diversity from 1978 to 2012, for the contiguous US on a county level basis. We used Shannon diversity indices expressed as effective number of crop species (ENCS) to quantify crop diversity. We then evaluated changes in county-level crop diversity both nationally and for each of the eight Farm Resource Regions developed by the National Agriculture Statistics Service. During the 34 years we considered in our analyses, both national and regional ENCS changed. Nationally, crop diversity was lower in 2012 than in 1978. However, our analyses also revealed interesting trends between and within different Resource Regions. Overall, the Heartland Resource Region had the lowest crop diversity whereas the Fruitful Rim and Northern Crescent had the highest. In contrast to the other Resource Regions, the Mississippi Portal had significantly higher crop diversity in 2012 than in 1978. Also, within regions there were differences between counties in crop diversity. Spatial autocorrelation revealed clustering of low and high ENCS and this trend became stronger over time. These results show that, nationally counties have been clustering into areas of either low diversity or high diversity. Moreover, a significant trend of more counties shifting to lower rather than to higher crop diversity was detected. The clustering and shifting demonstrates a trend toward crop diversity loss and attendant homogenization of agricultural production systems, which could have far-reaching consequences for provision of ecosystem system services associated with agricultural systems as well as food system sustainability.

## Introduction

Ecologists recognize biodiversity as integral to ecosystem function, affecting factors such as net productivity, ecosystem services, and resilience to perturbations [1]. However, most biodiversity research has focused on natural areas [2], despite the large spatial footprint of agricultural production. In the contiguous United States cropland occupies 408 million acres (165 million ha), or 22% of the land base, and in some regions, such as the Northern Great Plains or Corn Belt, more than 50% of the land area is cropland [3].

Biodiversity in agricultural systems is associated with critical ecosystem processes such as nutrient and water cycling, pest and disease regulation, and degradation of toxic compounds such as pesticides [4]. Diverse agroecosystems are more resilient to highly variable weather resulting from climate change [5] and often hold the greatest potential for sustaining important ecosystem services such as natural pest control [6]. For example, Meehan et al. [7] suggested that agricultural landscape simplification may result in increased pest pressure and a resulting increased use of insecticides. Agricultural landscapes also impact desirable or beneficial species. For instance, landscape structure or the amount of heterogeneity in the landscape is an important consideration in butterfly diversity and species composition [8].

Additionally, diverse cropping systems generally provide more varied and therefore more nutritious and healthful food for humans [4,9]. Although modern agriculture has been extremely successful at meeting the food, feed and fiber needs of an expanding global population, current approaches may have reduced agroecosystem biodiversity [10]. Some authors have suggested that biodiversity has declined both in terms of diversity of crop species grown as well as diversity of naturally occurring biological organisms that inhabit agroecosystems [4, 6]. However, research documenting changes in crop species diversity is lacking.

Crop diversity encompasses several aspects, including crop species diversity, varietal diversity within crop species, and genetic diversity within crop species and varieties. One could also consider other temporal, spatial, biological, genetic, and socio-economic aspects of crop production [9, 11, 12] when seeking to define crop or cropping system diversity. Within a cropping system, reduced genetic diversity within individual crop species has garnered attention recently [11]. However, crop species diversity, or the number of different crop species grown within a geographic area, is equally important but has received less attention.

One exception to the lack of information on crop species diversity is a recent global synthesis of national-scale crop species diversity data [13]. Although this analysis focused on consumption rather than production, it showed a slight increase in crop species richness contributing to food supplies but also increased homogenization of food supplies (i.e., crop commodities) across all countries during the last 50 years [13]. But how has crop species diversity changed in the US during recent time periods? This question can be explored via USDA-NASS Census of Agriculture [14], which provides detailed county-level statistics about US agricultural production that are collected every five years. The consistency of the data and the fine spatial scale at which it is collected provide a powerful tool for analyzing crop diversity changes over time.

Our objective was to quantify temporal and spatial crop species diversity at the county level for the contiguous US. We utilized the USDA-NASS Census of Agriculture [14] data from 1978 (the first year the data were digitized) to 2012 to develop Shannon diversity indices for individual counties at each time period. Because of the scope of our analyses and the enormous amount of data involved, we only included digitized datasets. We hypothesized that 1) crop diversity would change over time and 2) patterns of change would differ among geographic regions. For instance, anecdotal reports suggested that crop diversity likely declined in areas dominated by corn and soybean production; however, other factors such as adoption of no-till and consumer demand for new food crops could have increased crop species diversity. Our goal was to conduct analyses that would provide means to visualize and readily comprehend trends embedded in a massive dataset, recognizing that the reasons for various trends and changes are myriad and vary among numerous production regions.

## Materials and Methods

### Computation of Effective Number of Crop Species

We accessed the USDA-NASS database to obtain US Census of Agriculture data quantifying numbers of acres devoted to every recorded crop species grown in the contiguous US on a county-level basis during 1978, 1982, 1987, 1992, 1997, 2002, 2007, and 2012. Although the scale (county level) of our analysis is not as fine as that permitted by examining land cover data available through the NASS Cropland Data Layer, cover data for the entire Continental US have only been available since 2008. Accordingly using Census data permitted a wider temporal range for our analysis.

We computed crop species diversity for each county using the Shannon diversity index (SDI) expressed as:

$$SDI=\sum_{\text{i}=1}^{\text{s}}{\text{p}}_{\text{i}}{\;\text{ln}\;\text{p}}_{\text{i}}$$ (1)

Where *p*_*i*_ is the proportion of harvested area for crop i or the crop group. We specifically chose the Shannon index because it is more sensitive to rare species in terms of species richness as opposed to Simpson’s index, which is more responsive to dominant species, or descriptive of evenness [15]. According to Nagendra [16], the occurrence of opposite responses for these two indices range from 4 to 6%, depending on species richness. The resultant Shannon indices were subsequently expressed as effective number of crop species (ENCS), computed as:

$$ENCS={\text{e}}^{\text{-SDI}}$$ (2)

ENCS is an easily interpreted index of crop species diversity [15], with a value representing an estimate of the number of crops dominating production in a particular county. For example, a county producing 10 crops with each crop accounting for 10% of the acreage would have an ENCS = 10, whereas a county producing 10 crops with only one crop occupying 91% of the acreage, and the other nine crops each occupying 1% of the total acreage would have an ENCS = 1.65. Hereafter, we use ‘crop diversity’ to denote crop species diversity as quantified by ENCS.

To standardize data, crop aggregations used in the 1978 census were adopted for all census years, except for the addition of new crops. For example, wheat data collected from 1978 to 1992 were placed in one category, ‘wheat for grain.’ Subsequent censuses added categories such as durum, winter wheat, and non-specified wheat for grains. For our analyses, all wheat crops were collectively lumped as ‘wheat for grain,’ which may have underestimated crop species diversity in counties where both spring and winter wheat were produced. Also, where possible, we included unpublished data in our analyses. Unpublished data are data collected via the Census of Agriculture using the same approach as for published data, but withheld from publication because of privacy issues. For instance, if a county only had one wheat producer, then making wheat production data available for that county compromised the privacy of that one producer. The unpublished data was only a small fraction of the total dataset, but including it provided a more complete analysis. With the addition of the unpublished data, the database accounted for more than 90 percent of the crop land area for each state, and more than 99 percent of the total crop land area at the national level.

### Spatial and Statistical Analyses

**Fig 1:**
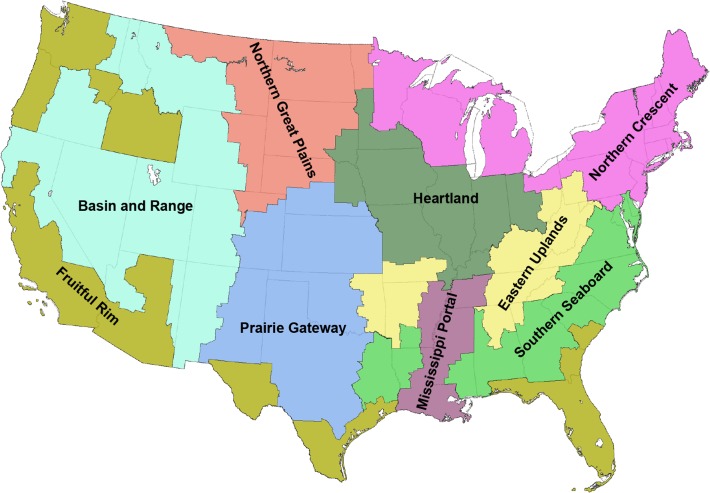
Farm Resource Regions of the US. Adapted from USDA-ERS [11].

To aggregate counties for discussion, we adopted Farm Resource Regions (Fig 1), as defined by the USDA Economic Research Service [17]. The use of Farm Resource Regions clusters counties with historically similar cropping practices, cutting across state boundaries and allowing crop diversity to be aggregated at the regional level.

Data were analyzed using spatial autocorrelation implemented using Gi and Gi* statistics, introduced in Ord and Getis [18] for the study of local pattern in spatial data within ArcGIS 10 (ESRI, Redmond, CA, USA) software environment. Nonbinary weights were allowed and statistics were related to Moran's autocorrelation statistic, I, which when not equal to zero indicates random placement and when equal to zero indicates clustering [19]. Correlations between nearby values of statistics were derived and verified by simulation. A Bonferroni correction was used to approximate significance levels when testing extreme values from the set of statistics. Hot spot analysis was implemented to detect spatial clustering of low and high ENCS values, designating them as hot and cold spot areas, respectively. This method utilizes the Moran’s I index and has been implemented successfully in hotspot identification of pollution [20] and soil carbon [21], among others.

To separate mean ENCS values among census years within each ERS region and among ERS regions within years, analysis of variance tests were conducted using PROC Mixed (SAS 9.3, Cary, NC), with ‘year’ and ‘ERS region’ treated as fixed effects in each of the respective models. Prior to conducting ANOVA tests, Levene’s test was performed to assess variance homogeneity, and the Shapiro-Wilk test was performed to assess normality. No transformations of data were necessary to conform to the assumption of ANOVA. When overall F-tests were significant, Tukey’s HSD was used to separate means. A significance level of *P* < 0.05 was used.

## Results and Discussion

The NASS Census of Agriculture data we analyzed allows us to provide a spatial and temporal estimate of diversity at the county level across the US. However, agriculture is complex and each Farm Resource Region has unique characteristics. Therefore, we did not intend to provide an exhaustive investigation for all reasons underlying the trends we uncovered, although we did provide a few detailed insights into trends noted in our home region, the Northern Great Plains. Additionally, the Census Data we analyzed do not contain information needed to assess temporal crop species diversity (i.e., the diversity of crop rotations over time). However, the diversity of crop species grown within a county or region likely correlates with temporal diversity because, during each year, the diversity of crop species that comprise a typical rotation would likely be represented.

**Fig 2:**
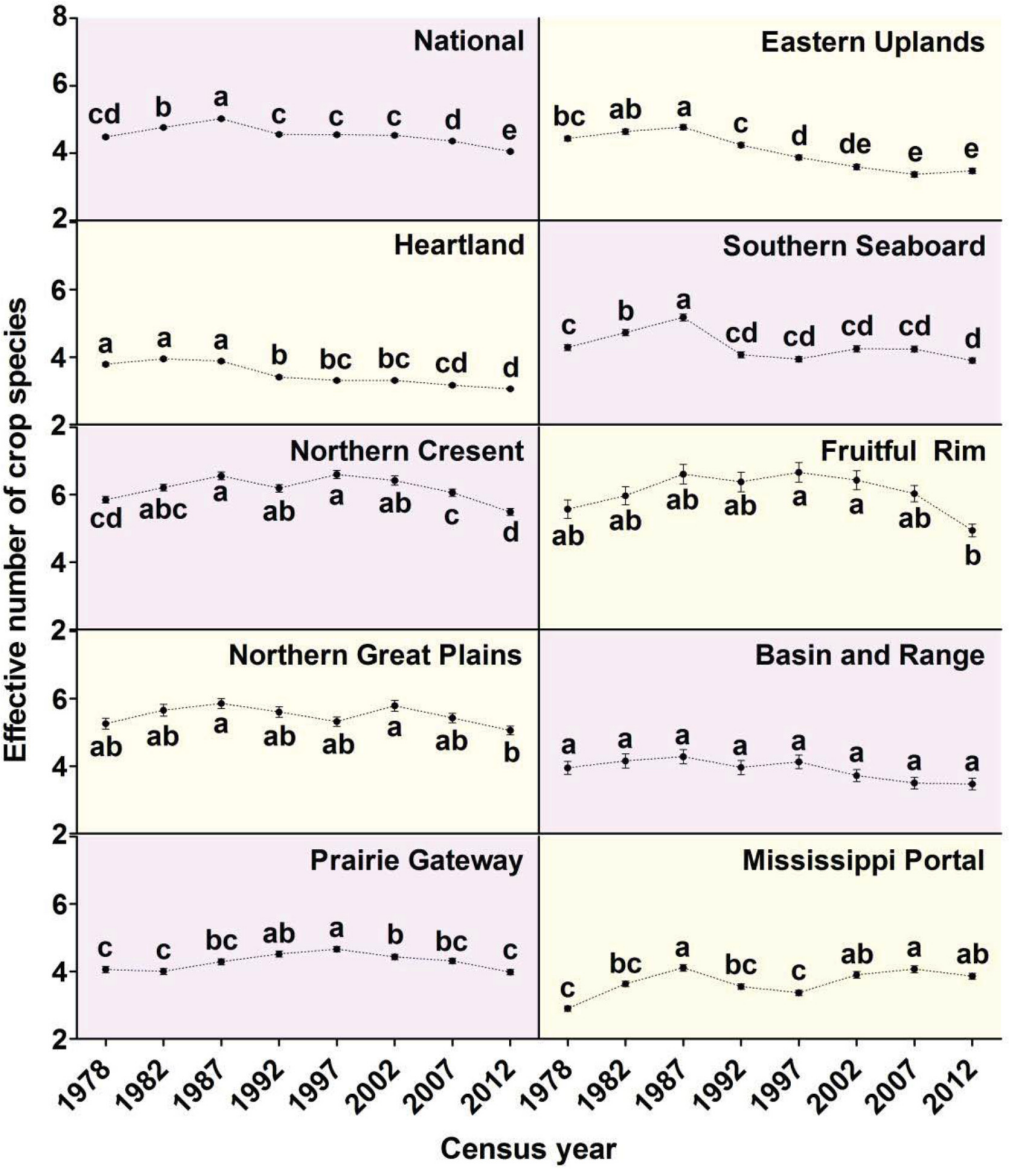
Mean effective number of crop species (ENCS) at the county level for each ERS Farm Resource Region (Eastern Uplands, Heartland, Southern Seaboard, Northern Crescent, Fruitful Rim, Northern Great Plains, Basin and Range, Prairie Gateway, and Mississippi Portal) and on a national basis (National). The ENCS was calculated from US Census of Agriculture data which was collected every five years from 1978 to 2012. Mean ENCS values are denoted by solid black circles, with error bars representing standard errors of the mean. Within each individual ERS Farm Resource Region and also at the national level, mean ENCS values for each census year labeled with different lowercase letters were significantly different according to Tukey’s HSD test (*P*<0.05).

Crop diversity (ENCS) at the national level increased from from1978 to 1987 (Fig 2). However, since 1987, crop diversity has decreased and crop diversity in 2012 was significantly lower than in 1978. Crop diversity in 1992 was similar to 1978 but by 2012, crop diversity was significantly lower than any other census period (Fig 2). Several factors may have contributed to the changes in crop diversity over the 35 years of the dataset. Expansion of simplified cotton-based or corn/soybean production systems has been driven by technological improvements [22]. Market forces can also shift diversity. For example, U.S. ethanol policy and rising demand for U.S. soybeans in China have increased corn and soybean acreage at the expense of wheat and other crops [23]. At the same time, high crop prices have resulted in perennial grasslands, such as those enrolled in the Conservation Reserve Program, being shifted to annual crop production [24].

However, not all Resource Regions followed the national pattern. Three of the nine Farm Resource Regions had lower crop diversity in 2012 compared to 1978, similar to the national trend. However, crop diversity in five of the Farm Resource Regions was the same in 2012 as in 1978, and crop diversity was higher in 2012 than in 1978 for the Mississippi Portal Region (Fig 2).

**Fig 3:**
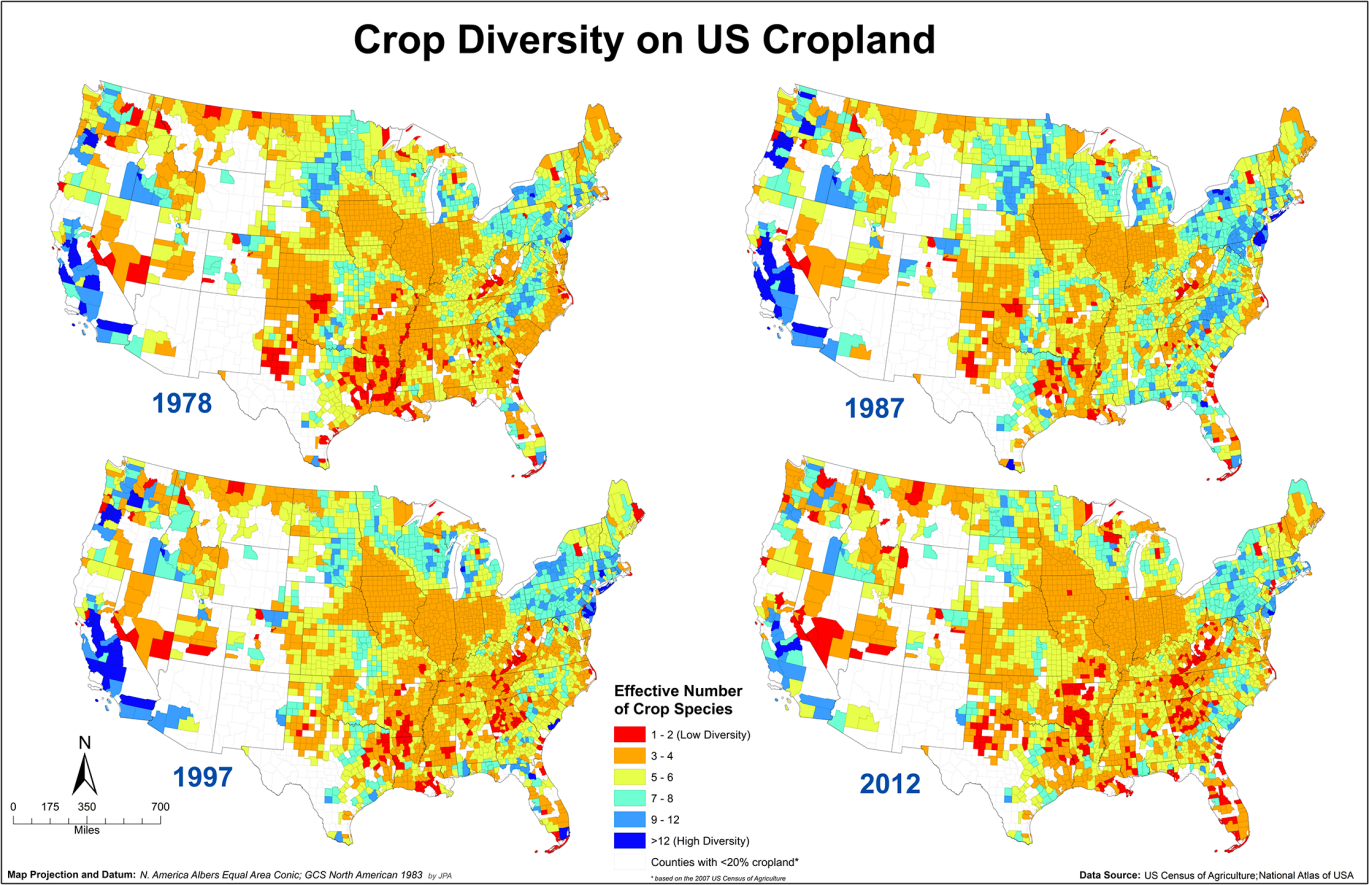
Crop species diversity as effective number of species in 1978, 1987, 1997 and 2012 on a county level basis for the contiguous US. The hotter colors (red hues) indicate lower ENCS values (low crop diversity) while colder colors (blue hues) indicate higher ENCS values (high crop diversity). Maps showing crop diversity for all Census years are available in S1 Fig.

The Heartland Resource Region had the lowest crop diversity in seven out of the eight census years (Table 1). The Heartland Resource Region, comprising most of the historical Corn Belt, is characterized by the greatest number of farms, largest production of cash grains, and highest production value of any of the Farm Resource Regions (Table 2) [25]. The productive soils and favorable climate of the Heartland Region allow producers to focus on high yielding corn/soybean systems. Improvements in genetics and agronomic practices have allowed corn and soybean yields to climb steadily over the past 40 years. However, continued increases may be difficult to achieve [22]. Similar dynamics occurred in the Eastern Uplands region, where crop diversity was significantly lower in 1997 compared to 1978 and continued to decline until 2012 (Fig 2). Decreasing crop diversity in eastern Ohio, portions of Kentucky and Tennessee and the Missouri, Arkansas and Oklahoma portions of the Eastern Uplands contributed to this decline (Fig 3).

**Table 1:** County level mean ± standard error effective number of crop species (ENCS) for each ERS Farm Resource Region (calculated using US Census of Agriculture data collected during 1978, 1982, 1987, 1992, 1997, 2002, 2007, and 2012.

	Year of the Census			
Region	1978	1982	1987	1992
Heartland	3.79±0.05d	3.95±0.05c	3.88±0.05d	3.41±0.05d
Northern Crescent	5.85± 0.10a	6.21 ± 0.10a	6.56 ± 0.12a	6.19 ± 0.11a
Northern Great Plains	5.26±0.16ab	5.66±0.18a	5.86±0.15b	5.61 ± 0.16a
Prairie Gateway	4.06±0.09cd	4.00±0.09c	4.29±0.09cd	4.52±0.09b
Eastern Uplands	4.43±0.07c	4.64±0.08b	4.77±0.08bc	4.24±0.07bc
Southern Seaboard	4.29±0.09c	4.73±0.09b	5.18±0.10b	4.07±0.09c
Fruitful Rim	5.57±0.27ab	5.97±0.27a	6.61±0.29a	6.38±0.29a
Basin and Range	3.95±0.19cd	4.16±0.21bc	4.28±0.21cd	3.96±0.21bcd
Mississippi Portal	2.90±0.08e	3.63±0.08c	4.11±0.10d	3.55±0.08cd
Region	1997	2002	2007	2012
Heartland	3.31± 0.04e	3.31±0.04d	3.17±0.04d	3.06±0.03e
Northern Crescent	6.60±0.12a	6.42±0.14a	6.06±0.11a	5.49±0.10 a
Northern Great Plains	5.32±0.14b	5.79±0.16a	5.43±0.14a	5.06±0.13ab
Prairie Gateway	4.66±0.08c	4.43±0.08c	4.31±0.08b	3.98±0.08c
Eastern Uplands	3.87±0.08d	3.59±0.08d	3.37±0.08d	3.47±0.08d
Southern Seaboard	3.94±0.08d	4.25±0.09c	4.24±0.09b	3.90±0.08c
Fruitful Rim	6.66±0.29a	6.43±0.28a	6.03±0.24a	4.94±0.19b
Basin and Range	4.13±0.20cd	3.72±0.18cd	3.50±0.17cd	3.47±0.17cde
Mississippi Portal	3.37±0.08de	3.90±0.10c	4.07±0.10c	3.86±0.09 cd

Within each year, differences among regional ENCS values are denoted by differing lowercase letters (P<0.05).

**Table 2:** Major commodities, percent of total US production, and numbers of farms for each Farm Resource Region during 2007. Major commodities were the two commodities with the greatest percent of US value of production for each resource region. The percentage of the total US production value for each of the commodities is given in parenthesis. Percent of total production is the percentage of US total production produced by a given Farm Resource Region. Number of farms for each resource region is given in the thousands. Data adapted from Hoppe and Banker [25].

Farm Resource Region	Major Commodities		Percent of Total US Production Value	Number of Farms in Thousands
Heartland	Cash Grains	(54%)	26%	436.6
	Hogs	(70%)		
Northern Crescent	Dairy	(33%)	11%	318.6
	High Value Crops^a^	(11%)		
Northern Great Plains	Cash Grains	(11%)	6%	99.4
	Beef	(9%)		
Prairie Gateway	Beef	(41%)	16%	315.5
	Cotton	(39%)		
Eastern Uplands	Tobacco	(20%)	6%	345.6
	Poultry	(23%)		
Southern Seaboard	Tobacco	(64%)	9%	242.5
	Poultry	(44%)		
Fruitful Rim	High Value Crops	(20%)	20%	261.9
	Dairy	(37%)		
Basin and Range	High Value Crops	(4%)	3%	89.9
	Beef	(4%)		
Mississippi Portal	Cotton	(22%)	3%	86.8
	Cash Grains	6%)		

^a^ High value crops are vegetable, fruits and tree nuts, nursery and greenhouse production.

Most other Farm Resource Regions in the US maintained more or less steady crop diversity from 1978 to 2012 (Fig 2). For example, the Prairie Gateway region, a relatively dry region that produces wheat and a significant portion of US cotton (Table 2), but is less suitable for corn/soybean production remained stable in terms of crop diversity with a slight increase in the 1990s (Fig 2). The Basin and Range resource region had no significant changes in crop diversity over multiple census dates. The Basin and Range resource region has a large amount of federal land (Fig 3) and contributes approximately 3% of US agricultural production (Table 2). While the Basin and Range resource region does produce some high value horticultural or tree crops (Table 2), the large amount of federal land indicates the primary land use will be livestock grazing. Therefore we would expect little change in crop diversity in this region. The Fruitful Rim region, where the majority of US fruit and vegetable production is concentrated (Table 2), and the Northern Crescent, with a large dairy presence (Table 2), have maintained consistently high crop diversity over time (Table 1). An exception was in the 2012 census when crop species diversity declined substantially in both regions (Fig 2). The Mississippi Portal resource region was the only region with greater crop diversity in 2012 than in 1978 (Fig 2). Cotton is a major crop in this region (Table 2) and cotton acreage declined significantly over the last half of the 2000s [26]. The loss of cotton acreage may have encouraged producers to plant other crops.

Detection of crop diversity changes depends on the scale at which changes are analyzed. Regions where mean crop diversity did not change significantly may have experienced patchy changes in either direction that offset mean change for that region overall. For example, in the Northern Great Plains region between 1978 and 2012, crop diversity increased in central North Dakota counties but decreased in eastern counties of North and South Dakota (Fig 3). During this time period, conservation tillage and no-till rapidly increased in central and western North Dakota. No-till conserves soil water which 1) facilitates growing a broader range of crop species [27] and 2) reduces the incidence of fallow in wheat/fallow rotations. The adoption of no-till might have allowed producers to expand their crop portfolio to take advantage of the conserved soil water, and the diverse crop rotations likely also helped to control weed and disease cycles, thus facilitating the added diversity. However, in the eastern Dakotas, which receive greater precipitation but still have relatively cool growing conditions, genetic improvements appear to have driven the shift to corn and soybean production. Therefore, crop diversity increases in central North Dakota may have offset losses in the eastern Dakotas, giving the Northern Great Plains a flatter trend in crop diversity changes compared to the Heartland (Fig 2, Fig 3).

**Fig 4:**
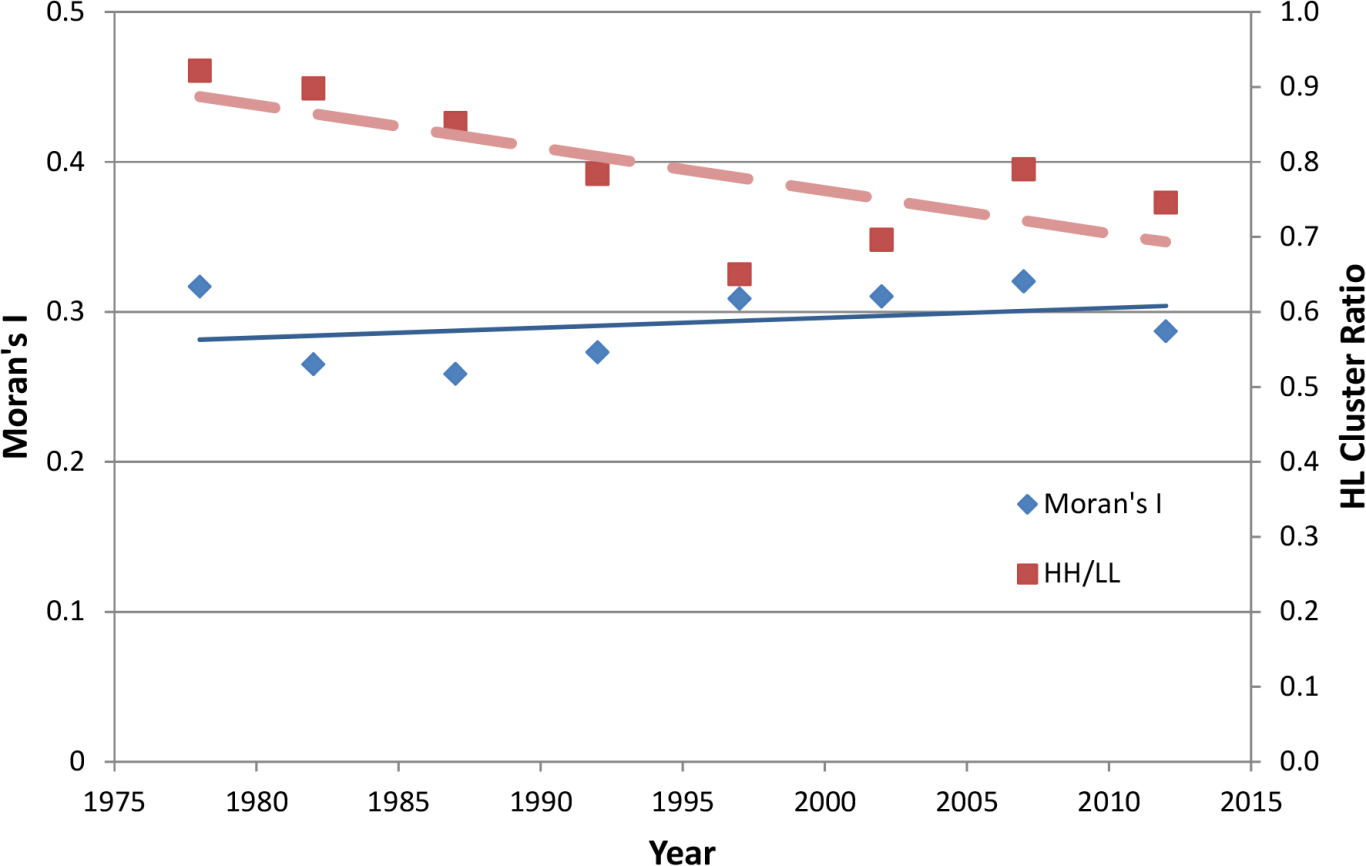
Measures of geospatial clustering, Moran’s I and High-Low Cluster Ratio over the period of analysis. Moran’s I value of 1.0 indicates very strong clustering of low or high ENCS values (Moran’s I = -1.0 indicates random occurrence). HL Cluster Ratio, derived from H/L Clustering analysis, indicates the number of clustered counties with high ENCS in relation to the number of clustered counties with low ENCS values. An HL cluster ratio of 1.0 indicates an equal number of clustered counties with high and low ENCS values, and a ratio approaching zero indicates a relatively large number of clustered counties with low ENCS than those with high ENCS.

Spatial autocorrelation indicated clustering into areas of low and high crop diversity. This was based on significant increases in Moran’s I value since 1978 (Sen’s slope = 0.001) (Fig 4). This indicates that areas with clusters of low crop diversity are becoming more pronounced or evident over time, much like clusters of high crop diversity. The increases in Moran’s I also indicate that areas with high and low diversity are becoming increasingly polarized. This may suggest that certain regions have over time become more focused on particular crop rotations, such corn/soybean, because such specialization has led to increased economic gain. To further examine spatial trends, we analyzed the ratio of counties with clusters of high values against low values, or HL cluster ratio. There was a downward trend (Sen’s slope = -0.009) in the HL cluster ratio (Fig 4). The HL cluster ratio is less than one, which indicates more counties are in clusters with low crop diversity than in county clusters with high crop diversity (Fig 4). The downward trend indicates that counties shifting from high to low crop diversity outnumber the counties shifting from low to high diversity.

**Fig 5:**
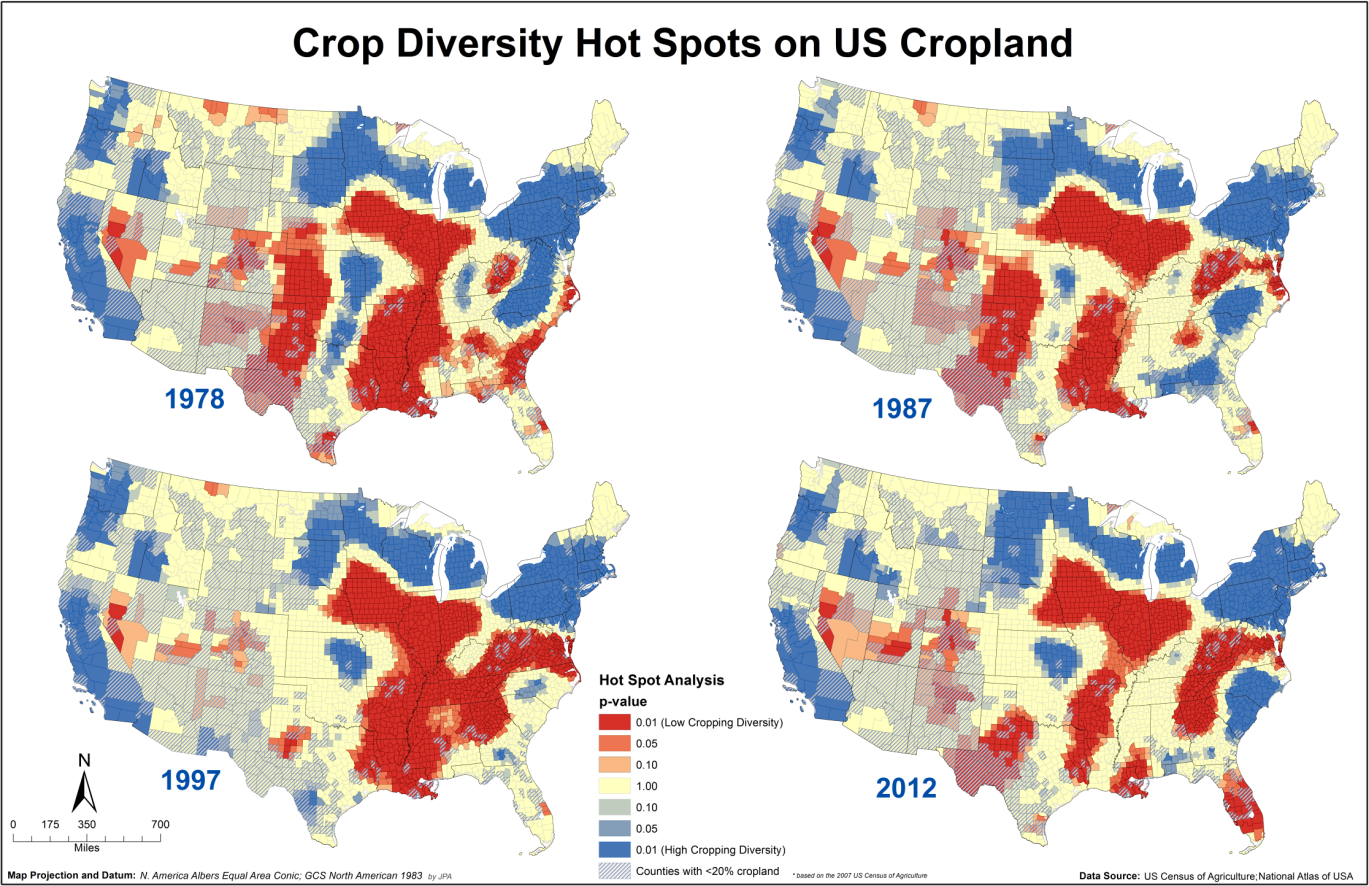
Crop species diversity hot spots based on the geostatistical analysis of the ENCS values for 1978, 1987, 1997 and 2012. Hot (red hues) spots are areas with significant clustering of counties with low ENCS values (low cropping diversity) and cold (blue hues) spots are clustering of counties with high ENCS values (high cropping diversity). Maps showing hot spot analysis for all Census years are available in S2 Fig.

The clustering into areas of high and low diversity can be visualized using hot spot analysis (Fig 5). Regions with low crop diversity extended from the Heartland Resource Region south to the Gulf of Mexico and also Eastern Uplands Resource Region to the Atlantic Ocean (Fig 5). The Northern Crescent Resource Region, portions of the Northern Great Plains and the Pacific Coast were areas with high crop diversity (Fig 5). In 1978, parts of the Atlantic Coast from Georgia to North Carolina, which included portions of the Southern Seaboard and Fruitful Rim Resource Regions, occupied areas of relatively low crop diversity. However, by 2012, this area was a cluster of relatively high crop diversity (Fig 5) mainly because of high crop diversity in counties in North and South Carolina (Fig 3).

**Fig 6:**
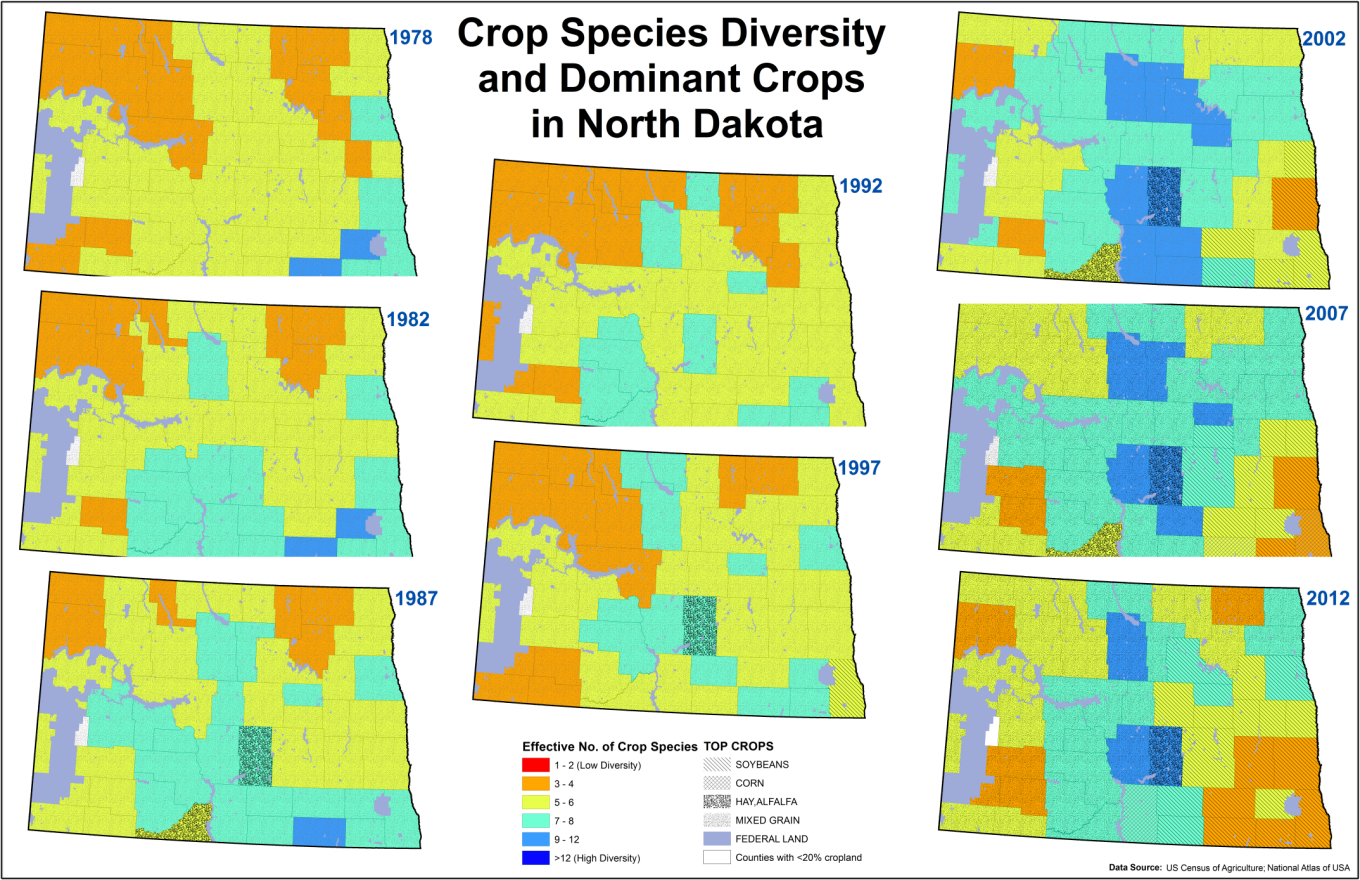
Crop Species Diversity and Dominant Crops in North Dakota. Solid colors indicate crop species diversity and federal lands while the overlayed pattern indicate the dominant crop for that county. The hotter colors (red hues) indicated lower ENCS values (low crop diversity) while colder colors (blue hues) indicate higher ENCS values (high crop diversity).

A great multitude of factors influence crop diversity at the county level; and these factors likely vary substantially according to region. Therefore, examining all the factors driving changes in crop species diversity is beyond the scope of our analysis. However, by evaluating trends in North Dakota, a state familiar to the authors, we hoped to illustrate some potential reasons for changes in crop diversity in the Northern Plains. In general, precipitation in North Dakota decreases from east to west and temperature decreases from south to north. In 1978, cropping systems in North Dakota were dominated by mixed grain crops (i.e., wheat, barley etc.) and the counties with the highest diversity were located in the southeastern corner of the state (Fig 6). In the 1970s, crop/fallow was the dominant cropping system for spring wheat and other cereals [28] especially in the more arid western parts of the state. During the 1980s, the advent of no-till, which helped to conserve soil water, allowed producers to increase cropping intensity by growing crops every year, rather than allowing for a fallow year. However, in the 1990s, fusarium head blight (*Gibberella zeae* (Schwein.) Petch), a fungus affecting wheat and barley, had a tremendous economic impact on small grain producers in North Dakota as well as surrounding states [29]. Producers, who had already began to grow more crops, needed alternatives to traditional small grains. Crop diversity began to increase, especially in the central part of the state, which was the mid-point for the precipitation gradient [30]. In the 2002 Census, soybeans, a crop associated with the Heartland Resource Region, became the largest crop in some counties in the eastern and southeastern corner of the state. There was a concomitant decline in crop diversity (Fig 6). In the 2012 Ag Census, the number of counties with soybeans as the dominant crop increased yet again while crop diversity continued to decrease.

The increase in soybean production cannot be linked directly to declines in crop diversity in the southeastern part of North Dakota. However, as detailed above, improvements in genetics and technology, along with rising demand and increased crop prices, have made corn and soybean a profitable combination for many producers. Not only have these systems been profitable in the past, the lack of complexity also decreases management intensity, which provides attractive labor savings for producers [31].

## Conclusions

The results of our analyses of US Census of Agriculture crop production data indicate crop species diversity declined in the US from 1978 to 2012, but that changes in crop diversity varied between and within regions. Potentially as interesting as the decline in US crop diversity are the differing trends among Farm Resource Regions. These differences highlight the multiple forces that impact farmer selection of specific crops.

Croplands comprise approximately 22% of the total land base in the lower 48 states. Therefore, changes in crop species diversity could have a substantial impact, not only on agroecosystem function, but also on the function of surrounding natural and urban areas. Croplands are unique ecosystems in that typically they are replanted annually. This means that, theoretically, crop species diversity can change relatively rapidly, so the potential for swift positive change is considerable, unlike in natural ecosystems. As discussed above, numerous important ecosystem benefits accrue from diverse cropping systems. However, the decline in crop species diversity appears to be ongoing, regardless of the well-documented benefits of diversity.

Modern farming is a complex and capital-intensive endeavor. In the US, during the twentieth century, increases in the value of human labor, changes in agricultural policies, and development of agricultural technology led to increased specialization and scale of production [32]. Farmers act in ways that best enhance their well-being, and therefore the adoption of less-diverse cropping systems appears to have benefitted farmers, both economically and socially. Unfortunately, many of the ecosystem benefits of diverse cropping systems are external to the economic accounting of farmers and are thus likely ignored [12].

However, future challenges may reverse the steady declines in crop species diversity within agricultural production systems. For instance, one important consequence of increased crop homogeneity is the potential for yield instability with anticipated increased unpredictability in weather patterns associated with climate change. Diverse cropping systems tend to increase farmers’ chances of encountering favorable conditions while decreasing the probability of widespread crop failures (i.e., risk is spread across a number of different ventures, or crops that require different weather conditions). Recent research results based on long-term data collected in Ontario, Canada, demonstrated that, compared to simple corn/corn and corn/soybean cropping systems, more diverse systems that included a small grain such as wheat, or under-seeding with a cover crop such as red clover, produced more stable yields over a 31 year period [33]. Increased crop diversity, combined with reduced tillage, also provided increased corn and soybean yields during dry hot years.

Increasing crop diversity has long been recognized as a method to reduce economic risk in agriculture and greater crop diversity has been linked to increased and more stable farm income in the wetter parts of the Canadian prairie [34] and other agricultural areas [35]. However, this relationship is complex because policies aimed at stabilizing revenues by supporting a particular crop can reduce diversity by uncoupling crop diversity and risk management [36]. Nevertheless, in the face of escalating challenges presented by climate change, policy changes that encourage crop diversity may be needed to support efforts to ensure future food security [37].

## Supporting Information

S1 Fig Crop species diversity as effective number of species for all of the Census of Agriculture years between 1978 and 2012. The hotter colors (red hues) indicate lower ENCS values (low crop diversity) while colder colors (blue hues) indicate higher ENCS values (high crop diversity).
(TIF)

S2 Fig Crop species diversity hot spots based on the geostatistical analysis of the ENCS values for all of the Census of Agriculture years between 1978 and 2012. Hot (red hues) spots are areas with significant clustering of counties with low ENCS values (low cropping diversity) and cold (blue hues) spots are clustering of counties with high ENCS values (high cropping diversity).
(TIF)

## References

[1] Duffy JE. Why biodiversity is important to the functioning of real-world ecosystems. Front. Ecol. Environ. 2009;7: 437–444.

[2] Tscharntke T, Klein AM, Kruess A, Steffan-Dewenter I, Thies C. Landscape perspectives on agricultural intensification and biodiversity-ecosystem service management. Ecol. Lett. 2005;8: 857–874.

[3] Nickerson C, Morehart M, Kuethe T, Beckham J, Ifft J, Williams R. Trends in U.S. Farmland Values and Ownership. 2012 USDA-ERS Econ. Info. Bull. Num. 92.

[4] Altieri MA. The ecological role of biodiversity in agroecosystems. Agric. Ecosys. Environ. 1999; 74: 19–31.

[5] Lin BB. Resilience in agriculture through crop diversification: adaptive management for environmental change. Bioscience. 2011;61: 183–193.

[6] Bianchi FJ, Booij CJ, Tscharntke T. Sustainable pest regulation in agricultural landscapes: a review on landscape composition, biodiversity and natural pest control. Proc. R. Soc B: Biological Sciences 2006; 273: 1715–1727. 16790403 10.1098/rspb.2006.3530 PMC1634792

[7] Meehan TD, Werling BP, Landis DA, Gratton C. Agricultural landscape simplification and insecticide use in the Midwestern United States. Proc. Natl. Acad. Sci. 2011; 108: 11500–11505. 10.1073/pnas.1100751108 21746934 PMC3136260

[8] Weibull AC, Bengtsson J, Nohlgren E. Diversity of butterflies in the agricultural landscape: the role of farming system and landscape heterogeneity. Ecography. 2000; 23:743–750.

[9] Frison EA, Cherfas J, Hodgkin T. Agricultural biodiversity is essential for a sustainable improvement in food and nutrition security. Sustainability 2011; 3: 238–253.

[10] Benton TG, Vickery JA, Wilson JD. Farmland biodiversity: is habitat heterogeneity the key? Trends Ecol. Evol. 2003; 18:182–188.

[11] Hajjar R, Jarvis DI, Gemmill-Herren B. The utility of crop genetic diversity and biotic similarity in maintaining ecosystem services. Agric. Ecosys. Environ. 2008; 123:261–270.

[12] Jackson LE, Pascual U, Hodgkin T. Utilizing and conserving agrobiodiversity in agricultural landscapes, Agriculture, Ecosystems & Environment, Volume 121, Issue 3, 7 2007, Pages 196–210

[13] Khoury CK, Bjorkman AD, Dempewolf H, Ramirez-Villegas J, Guarino L, Jarvis A, et al Increasing homogeneity in global food supplies and the implications for food security. Proc. Natl. Acad. Sci. 2014; 111:4001–4006. 10.1073/pnas.1313490111 24591623 PMC3964121

[14] agcensus.usda.gov [Internet]. Washington, DC. USDA- National Agricultural Statistics Service; c2012 [updated 2014 Apr 28; cited 2015, Apr 9]. Available: http://www.agcensus.usda.gov.

[15] Gotelli NJ, Chao A. Measuring and estimating species richness, species diversity, and biotic similarity from sampling data In: Levin SA, editor. Encyclopedia of Biodiversity 5, Waltham, MA: Academic Press; 2013 pp. 195–211.

[16] Nagendra H. Opposite trends in the response for the Shannon and Simpson indices of landscape diversity. 2002; Appl. Geogr. 22:175–186.

[17] Economic Research Service. 2000. USDA Ag. Info. Bulletin No. 760.

[18] Ord JK, Getis A. Local spatial autocorrelation statistics: distributional issues and an application. Geogr. Anal. 1995; 27: 286–306.

[19] Goodchild MF. 1986 Spatial autocorrelation (Vol. 47). Geo Books.

[20] Zhang C, Luo L, Xu W, Ledwith V. Use of local Moran's I and GIS to identify pollution hotspots of Pb in urban soils of Galway, Ireland. Sci. Total Environ. 2008;398: 212–221. 10.1016/j.scitotenv.2008.03.011 18440599

[21] McGrath D, CS Zhang. Spatial distribution of soil organic carbon concentrations in grassland of Ireland. Appl. Geochem. 2003;18:1629–1639

[22] Grassini P, Specht JE, Tollenaar M, Ciampitti I, Cassman KG. High-yield maize-soybean cropping systems in the US Corn Belt In: Sadras VO, Calderini D, editors. Crop Physiology: Applications for Genetic Improvement and Agronomy Amsterdam: Elsevier 2014 pp. 17–41.

[23] Jekanowski M, Vocke G. Crop outlook reflects near-term prices and longer term market trends. USDA Amber Waves 20 May 2013. Available: http://www.ers.usda.gov/amber-waves/2013-june/crop-outlook-reflects-near-term-prices-and-longer-term-market-trends.aspx#.VSf79pPveT8. Accessed 10 April 2015.

[24] Wright CK, Wimberly WC. Recent land use change in the Western Corn Belt threatens grasslands and wetlands. Proc. Natl. Acad. Sci. 2013; 110: 4134–4139. 10.1073/pnas.1215404110 23431143 PMC3593829

[25] Hoppe RA, Banker DE. Structure and Finance of US farms: Family farm report 2010 United States Department of Agriculture, Economic Research Service 2010. EIB No. 66.

[26] USDA-Economic Research Service. Cotton and wool background. Available: http://www.ers.usda.gov/topics/crops/cotton-wool/background.aspx.

[27] Peterson GA, Schlegel AJ, Tanaka DL, Jones OR. Precipitation use efficiency as affected by cropping and tillage systems. J. Prod. Agric. 1996; 9:180–186.

[28] Carr PM, Deibert EJ, Endres GJ, Henson RA, Jenks BM, Meyer DW, et al. Cropping systems research in North Dakota. Proc. Four State Cropping-Systems Symposium, 2002. Pierre, South Dakota March 7–8, 2002.

[29] Nganje WE, Bangsund DA, Leistritz FL, Wilson WW, Tiapo NM. Regional economic impacts of fusarium head blight in wheat and barley. Rev. Agric. Econ. 2004; 26:332–347.

[30] Hendrickson JR, Liebig MA, Sassenrath GF. Environment and integrated agricultural systems. Renewable Agric. Food Syst. 2008; 23:304–313.

[31] Hendrickson JR, Hanson JD, Tanaka DL, Sassenrath G. Principles of integrated agricultural systems: Introduction to processes and definition . Renewable Agric. Food Syst. 2008; 265–271.

[32] Bowman MS, Zilberman D. Economic factors affecting diversified farming systems. Ecology and Society 2013; 18:33.

[33] Gaudin ACM, Tolhurst TN, Ker AP, Janovicek K, Tortora C, Martin RC, et al Increasing crop diversity mitigates weather variations and improves yield stability. PLoS One. 2015; 10: e0113261 10.1371/journal.pone.0113261 25658914 PMC4320064

[34] Zentner RP, Wall DD, Nagy CN, Smith EG, Young DL, Miller PR et al Economics of crop diversification and soil tillage opportunities in the Canadian prairies. Agron. J. 2002;94: 216–230.

[35] Di Falco S, Perrings C. Crop genetic diversity, productivity and stability of agroecosystesm. A theoretical and empirical investigation. Scott. J. Polit. Econ. 2003; 50:207–216.

[36] Di Falco S, Perrings C. Crop biodiversity, risk management and the implications of agricultural assistance. Ecol. Econ. 2005; 44:459–466.

[37] Howden SM, Soussana JF, Tubiello FN, Chhetri N, Dunlop M, Meinke H. Adapting agriculture to climate change. Proceedings of the National Academy of Sciences of the United States of America, 2007; 104:19691–19696. 18077402 10.1073/pnas.0701890104 PMC2148359

