# Supplementary figures and images for: Crop Species Diversity Changes in the United States: 1978–2012
Source: PLoS One. 2015 Aug 26;10(8):e0136580. doi: 10.1371/journal.pone.0136580 (PMC4550420; doi:10.1371/journal.pone.0136580)

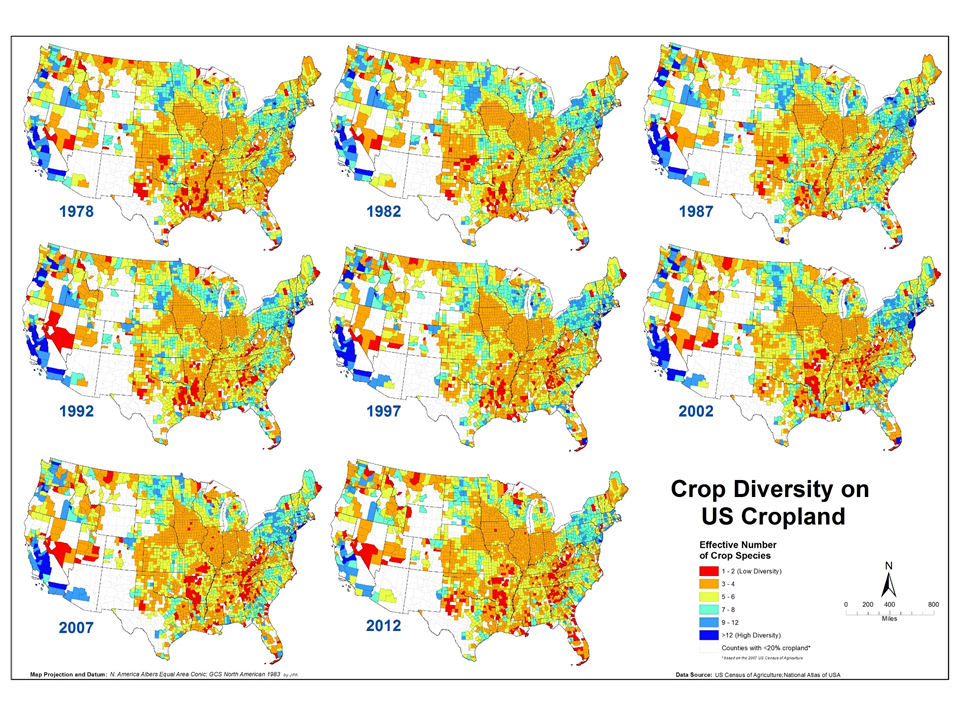

Supplement: S1 Fig — The hotter colors (red hues) indicate lower ENCS values (low crop diversity) while colder colors (blue hues) indicate higher ENCS values (high crop diversity). (TIF) [file pone.0136580.s001.tif]

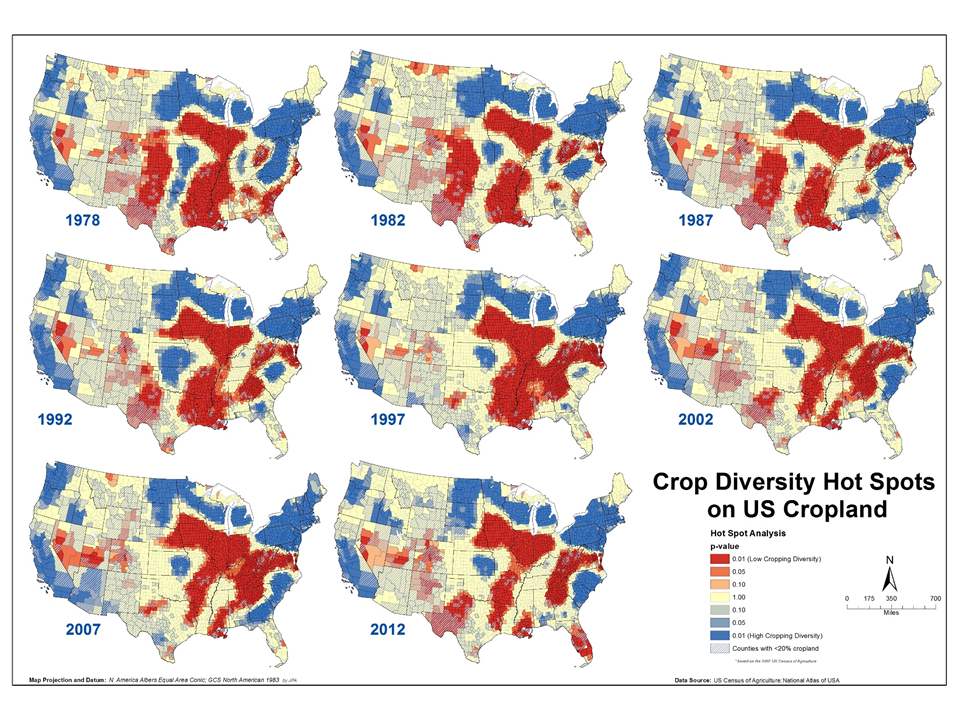

Supplement: S2 Fig — Hot (red hues) spots are areas with significant clustering of counties with low ENCS values (low cropping diversity) and cold (blue hues) spots are clustering of counties with high ENCS values (high cropping diversity). (TIF) [file pone.0136580.s002.tif]
